# Supplementary material for: Morphological Characteristics, Ultrastructure, and Chemical Constituents of the Endotesta in Ginkgo (Ginkgo biloba L.)
Source: Plants (Basel). 2023 Oct 13;12(20):3560. doi: 10.3390/plants12203560 (PMC10609943; doi:10.3390/plants12203560)
Supplement: Supplementary file 1 [file plants-12-03560-s001.zip › plants-2629358-supplementary.pdf]

Supplementary materials

Morphological, ultrastructure, and chemical constituents of the endotesta in *Ginkgo biloba* L.

Fangdi Li [a], Linying Zhao [a, b], Zhuolong Shen [a], Qirong Guo [a, *]

[a] Co-Innovation Center for Sustainable Forestry in Southern China, College of Forestry, Nanjing Forestry University, Nanjing, 210037, Jiangsu, China
[b] Suzhou Planning and Design Research Institute Co., Ltd., Suzhou, 215000, Jiangsu, China

***Correspondence:** Qirong Guo
qrguo@njfu.edu.cn

Table S1. Composition of amino acids in the endotesta of *Ginkgo biloba*

Name of amino acid	Amino acid contents (mg/g)
Aspartic acid (Asp)	4.95±0.07
Glutamic acid (Glu)	5.04±0.06
Serine (Ser)	3.28±0.04
Histidine (His)	1.28±0.07
Glycine (Gly)	3.43±0.06
Arginine (Arg)	1.90±0.08
Alanine (Ala)	3.26±0.08
Tyrosine (Tyr)	0.90±0.10
Cystine(Cys)	0.27±0.05
Proline(Pro)	3.48±0.20
Threonine (Thr)	2.57±0.11
Valine(Val)	2.87±0.12
Methionine (Met)	0.20±0.07
Isoleucine (Ile)	2.28±0.04
Phenylalanine (Phe)	1.85±0.06
Leucine (Leu)	3.67±0.06
Lysine (Lys)	2.54±0.07
The total content of amino acids (TAA)	43.79±1.09
The total content of essential amino acids (EAA)	15.99±0.40
The total content of non-essential amino acids (NEAA)	27.80±0.70
EAA/TAA (%)	36.52
EAA/NEAA (%)	57.51

Table S2. Fatty acid composition in the endotesta of *Ginkgo biloba*

Fatty acid composition		Molecular formula	Contents (ug/g)
Unsaturated fatty acid (UFA)	Palmitic acid	$C_{15}H_{31}COOH$	2828.89±12.65
	Linoleic acid	$C_{17}H_{35}COOH$	1867.21±14.24
	Elaidic acid	$C_{17}H_{33}COOH$	1284.79±17.61
	Dihomo-γ-linolenic acid	$C_{20}H_{35}COOH$	344.99±6.26
	Oleic acid	$C_{17}H_{33}COOH$	321.12±6.08
Saturated fatty acid (SFA)	Behenic acid	$C_{21}H_{43}COOH$	13581.51±384.74
	Lignoceric acid	$C_{23}H_{47}COOH$	6683.56±254.96
	Montanic acid	$C_{27}H_{55}COOH$	5500.77±99.14
	Cerotic acid	$C_{25}H_{51}COOH$	5345.90±99.14
	Melissic acid	$C_{29}H_{59}COOH$	1664.75±6.15
	Tricosanoic acid	$C_{22}H_{45}COOH$	1256.66±25.91
	Stearic acid	$C_{17}H_{31}COOH$	1113.44±10.56
	Pentacosanoic acid	$C_{24}H_{49}COOH$	998.12±35.39
	Arachidic acid	$C_{19}H_{39}COOH$	928.25±9.38
	Heneicosanoic acid	$C_{20}H_{41}COOH$	587.17±13.24
	Cis-9-Hexadecenoic acid	$C_{15}H_{29}COOH$	265.61±4.85
	Pentadecanoic acid	$C_{14}H_{29}COOH$	116.09±2.70
	15-Methyl-hexadecanoic acid	$C_{16}H_{33}COOH$	105.82±1.84

Table S3. Major vitamin content in the endotesta of *Ginkgo biloba*

Vitamins	Contents (μg/g)	Vitamins	Contents (μg/g)
L-ascorbic acid VC	44.72±0.61	Riboflavin VB_2	6.43±0.45
α-Tocopherol VE	17.22±0.59	Nicotinic acid VB_3	4.66±0.46
Cobalamin VB_{12}	16.17±0.16	Retinol VA	1.47±0.26
Thiamine VB_1	7.26±0.19		

Table S4. Content of major inorganic elements in the endotesta of *Ginkgo biloba*

Chemical element	Contents (mg/kg)	Chemical element	Contents (mg/kg)
Potassium (K)	11195.59±126.38	Boron (B)	35.095±1.12
Calcium (Ca)	3077.86±75.12	Aluminium (Al)	25.43±1.50
Zinc (Zn)	1055.96±1.02	Manganese (Mn)	11.75±0.53
Sulfur(S)	901.53±8.26	Strontium (Sr)	11.45±0.09
Magnesium (Mg)	805.48±26.27	Barium (Ba)	6.76±0.20
Phosphorus (P)	424.95±6.31	Copper (Cu)	2.97±0.08
Sodium (Na)	284.15±9.17	Chromium (Cr)	1.72±0.21
Iron (Fe)	83.11±4.13	Cobalt (Co)	1.28±0.18
